# Pharmacy refill adherence outperforms self-reported methods in predicting HIV therapy outcome in resource-limited settings

**DOI:** 10.1186/1471-2458-14-1035

**Published:** 2014-10-04

**Authors:** Raphael Z Sangeda, Fausta Mosha, Mattia Prosperi, Said Aboud, Jurgen Vercauteren, Ricardo J Camacho, Eligius F Lyamuya, Eric Van Wijngaerden, Anne-Mieke Vandamme

**Affiliations:** Department of Pharmaceutical Microbiology, Muhimbili University of Health and Allied Sciences, Dar es Salaam, Tanzania; Department of Microbiology and Immunology, Rega Institute for Medical Research, Clinical and Epidemiological Virology, KU Leuven - University of Leuven, B-3000 Leuven, Belgium; Ministry of Health and Social Welfare, Dar es Salaam, Tanzania; Centre for Health Informatics, Institute of Population Health, University of Manchester, Manchester, UK; Department of Microbiology and Immunology, Muhimbili University of Health and Allied Sciences, Dar es Salaam, Tanzania; Janssen Pharmaceutica, Beerse, Belgium; Centro de Malária e outras Doenças Tropicais and Unidade de Microbiologia, Instituto de Higiene e Medicina Tropical, Universidade Nova de Lisboa, Lisbon, Portugal; University Hospitals, University of Leuven, Leuven, Belgium

**Keywords:** Antiretroviral, Adherence validation, Questionnaires, Sensitivity and specificity, HIV-1, Tanzania, Africa

## Abstract

**Background:**

Optimal adherence to antiretroviral therapy is critical to prevent HIV drug resistance (HIVDR) epidemic. The objective of the study was to investigate the best performing adherence assessment method for predicting virological failure in resource-limited settings (RLS).

**Method:**

This study was a single-centre prospective cohort, enrolling 220 HIV-infected adult patients attending an HIV/AIDS Care and Treatment Centre in Dar es Salaam, Tanzania, in 2010. Pharmacy refill, self-report (via visual analog scale [VAS] and the Swiss HIV Cohort study-adherence questionnaire), pill count, and appointment keeping adherence measurements were taken.

Univariate logistic regression (LR) was done to explore a cut-off that gives a better trade-off between sensitivity and specificity, and a higher area under the curve (AUC) based on receiver operating characteristic curve in predicting virological failure. Additionally, the adherence models were evaluated by fitting multivariate LR with stepwise functions, decision trees, and random forests models, assessing 10-fold multiple cross validation (MCV). Patient factors associated with virological failure were determined using LR.

**Results:**

Viral load measurements at baseline and one year after recruitment were available for 162 patients, of whom 55 (34%) had detectable viral load and 17 (10.5%) had immunological failure at one year after recruitment. The optimal cut-off points significantly predictive of virological failure were 95%, 80%, 95% and 90% for VAS, appointment keeping, pharmacy refill, and pill count adherence respectively. The AUC for these methods ranged from 0.52 to 0.61, with pharmacy refill giving the best performance at AUC 0.61.

Multivariate logistic regression with boost stepwise MCV had higher AUC (0.64) compared to all univariate adherence models, except pharmacy refill adherence univariate model, which was comparable to the multivariate model (AUC = 0.64). Decision trees and random forests models were inferior to boost stepwise model.

Pharmacy refill adherence (<95%) emerged as the best method for predicting virological failure. Other significant predictors in multivariate LR were having a baseline CD4 T lymphocytes count < 200 cells/μl, being unable to recall the diagnosis date, and a higher weight.

**Conclusion:**

Pharmacy refill has the potential to predict virological failure and to identify patients to be considered for viral load monitoring and HIVDR testing in RLS.

**Electronic supplementary material:**

The online version of this article (doi:10.1186/1471-2458-14-1035) contains supplementary material, which is available to authorized users.

## Background

Widespread antiretroviral scale-up programs are taking place in resource-limited settings (RLS) [1]. Where resources are available, monitoring response to combination antiretroviral therapy (ART) by virological outcome is recommended because of its strong correlation with therapy success in HIV patients. At virological failure, therapy changes are then guided by genotypic resistance testing. However, sophisticated laboratory tests, such as virological monitoring and genotyping, are very expensive and not used in RLS [1–3]. Therefore, in RLS, clinical or immunological criteria are used to evaluate ART outcome. It has been shown that relying on such clinical and immunological criteria to change therapy can cause high levels of HIV drug resistance (HIVDR), compromising the next line of therapy [2, 3]. It is, therefore, important to identify affordable proxy markers for response to therapy in RLS. There is sufficient international evidence to support that adherence to combination ART is a major predictor of viral suppression [4, 5], HIVDR [6, 7], CD4 T lymphocytes count recovery [8] and survival [5, 8, 9].

Strategies to measure adherence to ART include the Medication Event Monitoring System (MEMS), pharmacy refill records, pill count, appointment keeping, adherence diaries, and interviewer-administered or self-report questionnaires [10–13]. Current objective methods of measuring adherence include MEMS, biologic measures, and pill counts [14, 15]. Each of these assessment methods is associated with certain strengths and drawbacks. MEMS is widely used in clinical studies, whereas pill count, self-report, and pharmacy refill are widely used in the context of HIV and AIDS clinical-care settings.

Self-report utilizing structured questionnaires is popular in RLS, due to its ease of use in busy settings, affordability, and low staff requirements. Self-report has consistently been correlated with viral load response to therapy and has been proposed as a robust and appropriate indicator of adherence [16, 17]. However, other documented studies showed that self-reports have low sensitivity and positive predictive value [18]. While self-reported adherence may be consistently correlated with important clinical outcomes, these relationships are generally modest at best, and the method continues to lack accuracy and precision. Self-reported adherence is known to overestimate adherence, due to recall bias and social desirability. Furthermore, the inaccuracy of self-reports is highlighted by the need to often dichotomize the highly-skewed data with cut-off points of 90 – 100% in most adherence analyses [17]. A combination of adherence strategies is recommended to improve the accuracy of this measurement [18].

In sub-Saharan Africa, adherence to ART has been touted to be equal to or even higher than levels in resource-rich settings (RRS) [19]. However, many adherence studies are based on non-objective self-report measurements and are not well validated against viral load measurement [20]. Therefore, it is still arguable whether these higher adherence reports reflect the actual situation. Moreover, treatment failures have been shown, even with self-reported high adherence [21]. It is important to acknowledge that self-reported adherence measures are far from robust. Its wide use and acceptance need further justification than the few selected studies that show correlation between this measure with other objective measures and with virological, clinical immunological, and survival outcome [16].

Pill count and appointment keeping adherence are among the early warning indicators (EWIs) recommended by the World Health Organization (WHO) as surrogate markers for emergence of HIVDR [22]. Manual pill count is a cheap and easy alternative to estimate dosages taken. However, it is limited by the intensive need of staff and the possibility of pill sharing or dumping by the patients. Appointment keeping can be easily abstracted from attendance records, but is only a subjective measure.

Another objective method that has higher potential is pharmacy refill adherence, which is commonly obtained in clinical care. It has been validated as a measure of ART adherence related to viral load [10]. Although pharmacy refill method is not constrained by the recall bias seen with self-report, its utility for predicting patient outcomes is poorly validated in RLS [23, 24].

Apparently, there is no single consensus tool to capture adherence to ART and no accepted gold standard to assess adherence especially in RLS [14, 15, 25, 26]. Moreover, studies show low or moderate correlation among methods, which can be attributed to the fact that they measure separate dimensions of adherence behaviour, that different non-adherence cut-off points have been established, or even to the limitations of the methods themselves. These issues result in varied non-adherence frequency measures throughout time and among diverse settings.

Validating adherence measurement against viral load is of utmost importance. An accurate and sensitive measure of adherence should give clinicians objective data to use during discussions on individuals’ non-adherence risk factors without causing shame or stigma to a patient [27]. The fear of stigma is a problem in non-adherence disclosure and can lead to over-reporting of adherence. The objective of the current study was to validate an instrument capable of measuring adherence to ART in HIV-infected adults by evaluating how such measurements can be used to predict virological outcome in Tanzania. Such an instrument can be used to select patients for viral load measurement and HIVDR testing, as a lower-cost alternative to generalized viral load follow-up.

## Methods

### Study design

**Figure 1:**
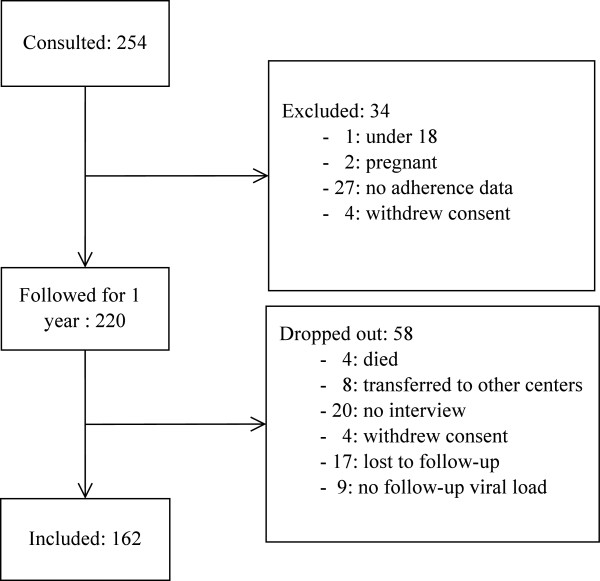
**Patients sampling flowchart.**

This was a single-centre prospective cohort study enrolling HIV-infected adult patients attending an HIV/AIDS Care and Treatment Centre (CTC) that provides ART at Amana District Hospital in Dar es Salaam, Tanzania, in 2010. Patients on ART are normally scheduled to return for antiretroviral (ARV) pick-ups at least once per month. During the visit, they also consult with the clinician after receiving adherence counselling from the nurses. CD4 T lymphocytes count measurements are taken at least once every six months. Two hundred and fifty-four patients were invited into the study during the months of May to July, 2010. Selection criteria were either starting ART or being on ART. Patients were conveniently sampled, as the study researcher continuously recruited patients who were referred by nurse counsellors, the first contacts at the clinic. Each day, 10-15 unselected patients were recruited into the study. Exclusion criteria were being younger than 18 years of age, pregnant, having opportunistic infections, or malignancy. Of the 254 patients recruited into the study, 34 (13.4%) were excluded from analysis for various reasons (see Figure 1). The remaining 220 provided adherence information and were followed for a period of one year. The herein presented study included 162 patients who completed a one-year follow-up and had their adherence, immunological, and virological outcome monitored over the entire period.

### Ethical issue

Issues pertaining to patient confidentiality, benefits and risks to participating patients, justice, rights and respect that the patients deserve were addressed by ethical clearance and informed consent. The study was approved by the Muhimbili University of Health and Allied Sciences (MUHAS) Research Ethics Committee. Only patients who were willing to participate in the study and who signed an informed consent were recruited into this study. Patient codes were used to de-link the patient data in databases. Patients did not receive any payments to motivate them to participate in the study.

### Virological and immunological outcomes

Virological and immunological outcomes were assessed at the one-year follow-up. These outcomes were dichotomized according to the following definitions: Virological failure was defined as having a viral load above the detection limit of 400 copies/mL; and immunological failure was defined according to the WHO guidelines as having (i) a CD4 T lymphocytes count of 100 cells/μl at six months post ART, (ii) a CD4 T lymphocytes count of equal to or less than CD4 pre-ART at six months on ART, or (iii) 50% reduction from the on-ART peak CD4 T lymphocytes count [28, 29].

### Adherence measurement

Adherence was measured using pharmacy refill, self-report, pill count, and appointment keeping methods, as described for each method. Adherences measurements were taken at four time points during a one-year follow-up, including at recruitment (zero), one, two, and 12 months after recruitment. Overall adherence for each method was the mean of the measurements taken at the four time points and this mean was considered in subsequent analyses.

### Pharmacy refill

Each refill period was identified as the interval between the last visit date and the scheduled refill date. Refill adherence was 100% if all pills during the scheduled refill period had been picked up on time. Refill percent values above 100 for patients who refilled earlier than scheduled were rounded to 100 percent. Refill adherence was not calculated on a monthly basis to reduce an error of a few additional pills left over at the end of each refill period. The calculation was based on cumulative sum of the days that a patient was late for ARV pick-up appointments in each month over the year, divided by the total number of days over all periods between pick-up periods in the year of study, resulting in the percentage of time the patient was without medication over the whole year.

### Self-report

The researcher for this study administered a self-report questionnaire to patients to assess missed dosages over the previous one month. During a one-year follow-up, self-report was used to determine adherence at recruitment (zero), one, two, and 12 months after recruitment. For patients who started ART at recruitment, the measurement at month zero was not available. Patient self-reported adherence was investigated using a validated study tool [26]. This validated study tool consists of two major sections: 1) the visual analog scale (VAS), which probed the percentage of doses taken in the previous month; and 2) it probes two questions from the Swiss HIV Cohort Study Adherence Questionnaire (SHCS-AQ) regarding frequency of missed doses and if a patient ever missed two consecutive doses (drug holiday) in the previous month. By definition, reporting of having missed at least one ARV dose or two consecutive doses in the month preceding the interview was scored as non-adherent [26, 30].

### Pill count

At each visit, pills remaining in bottles were counted and the proportion of these pills to the dispensed pills during the previous visit was calculated based on the dose and the number of days dispensed. The pill count adherence percent was obtained by dividing the number of pills consumed by the total number of pills at the beginning of the given interval and multiplied by 100.

### Appointment keeping

At this CTC, appointments were issued based on the period that the dispensed ARV stock would last, which usually this amounts to one month. Appointment keeping was deduced from the medical records and database. A patient visiting on the scheduled appointment was scored as 100% appointment adherent, whereas if an appointment was skipped entirely, it was scored as 0% adherence. For a delayed appointment, the proportions of days late (in% with respect to the total number of days between two visits) were subtracted from 100.

### Statistical analyses

Univariate analysis was done to explore a cut-off that gives a better trade-off between sensitivity and specificity, and a higher area under the curve (AUC), based on receiver operating characteristic (ROC) curve in predicting virological failure. For each method, we used a threshold cut-off beginning with 100% and decreasing to 50% in steps of 5. At the selected cut-off for pharmacy refill, VAS, pill count, and appointment keeping (a binary variable-expressed adherence, such that patients with adherence below the cut-off were classified as non-adherent, whereas the others were classified as adherent). The trade-off for sensitivity and specificity was analysed upon changing the adherence cut-off to predict virological failure at 12 months, which was dichotomized using two different viral load cut-off points: > 400 copies/mL or > 1,000 copies/mL. The 400 copies/ml virological cut-off was the detection limit of this study’s viral load measurement, while 1,000 represented a virologic cut-off widely used to perform genotyping. A model was also tested combining adherence and immunological outcome to predict virological failure. The performance criteria for all adherence measures were calculated with two-by-two tables and ROC analysis. Univariate logistic regression (LR) was used to determine patient baseline factors associated with virological outcome. Also, evaluation of the adherence models was done by fitting multivariate LR with stepwise functions (Akaike information criterion [AIC] and boost), decision trees, and random forests assessing extra-sample error via multiple 10-fold cross validation (MCV), repeated 50 times with random seeds. All models were analysed in terms of different performance (goodness-of-fit functions), specifically AUC, accuracy, true positive rate (sensitivity), and true negative rate (specificity). Performance distributions were compared pair-wise with a t-test adjusted for sample overlap (due to the MCV procedure) and multiple comparisons.

Kaplan Meier survival analysis was used to determine the difference between the time taken to increase the CD4 T lymphocytes count by 50 cells/μl or by 100 cells/μl between adherent and non-adherent patients. Furthermore, Cox regression analysis was done to adjust for confounding factors such as age, gender, and CD4 T lymphocytes count at baseline. Descriptive analyses, including median interquartile range (IQR) for numerical variables, frequencies, and proportions for categorical variables, and tested for association using Fisher and Chi-square tests for categorical variables, and Wilcoxon signed rank test for continuous values. The level of significance for all analyses was p < 0.05. All statistical analyses were performed using the R statistical package version 2.15.1 [31] and Weka open source software [32].

## Results

### Cohort information

A total of 162 (73.6%) out of the 220 patients followed for a one-year period had a virological outcome, an immunological outcome, and adherence data at the one-year time point and were included in this study (see Figure 1). The median IQR follow-up duration was 13 (11 - 13) months. Of the 220 patients, 49 (22.3%) were excluded because they could not be interviewed at the one-year follow-up. Four (1.8%) had died before the one-year end point; eight (3.6%) were transferred to other centres; 20 (9.1%) visited in the last month, but could not meet the interviewer; and 17 (7.7%) were likely to be lost to follow-up, as they had missed their appointments for at least three months. An additional nine (4.1%) patients were excluded from the study, due to lack of viral load measurement. There was no significant difference in terms of socio-demographic and social support characteristics, adherence measured by different methods, and immunological status between included and excluded patients (see Additional file 1). They also did not differ in duration of therapy or support for treatment. However, included patients were more likely to receive atripla or a once-daily single tablet regimen than the excluded group, while other therapy regimen distribution was not different. Also, included patients had a CD4 T lymphocytes count measurement performed significantly more often than the excluded group; eight (6 - 11) times compared to seven (4 - 11) times (p = 0.03).

Among the patients included in the study, the median IQR of ART exposure by the end of the observation period was 37 (30 - 48) months. This information was only available for patients with follow-up. With regard to duration of therapy at recruitment, there was no significance difference between included and excluded patients (p = 0.36).

### Performance of adherence models

With regard to adherence measurements, performance for various cut-off points to predict a viral load above 400 or above 1,000 copies/ml is shown in Table 1, Additional file 2, and Additional file 3. The 400 copies/ml virological cut-off was the study’s detection limit of viral load measurement, while 1,000 represents virologic cut-off widely used to perform genotyping. For SHCS-AQ, there are no cut-off points, as non-adherence is defined as in the methods. Evaluation of prediction by adherence measurements taken at different time points of follow-up and by overall adherence was similar (see Additional file 3). For the remainder of this paper, only overall adherence is considered. Prediction performance was better when predicting a viral load above 1,000 copies/ml (compared to above 400 copies/ml; see Additional file 2), but this viral load was not the clinically relevant one and was not analysed further. The optimal trade-off between highest AUC, acceptable sensitivity and specificity, and still being significantly predictive for virological failure (VL > 400 copies/ml) was 95%, 80%, 95% and 90% for VAS, appointment keeping, pharmacy refill, and pill count adherence respectively (see Table 1). At these adherence cut-off values, the proportion of adherent patients were 87.0%, 78.9%, 24.5%, and 54.3% for VAS, appointment keeping, pill count, and pharmacy refill adherence respectively. Whereas, for SHCS-AQ 71.6% of patients were adherent. For VAS, appointment keeping, pharmacy refill, and pill count adherence, the median IQR was 100 (98.33 - 100), 100 (84.33 - 100), 89.55 (86.23 - 100), and 78.83 (74.04 - 89.90) respectively. Combining adherence and immunological outcome improved the accuracy and sensitivity to predict virological failure, while specificity was not much worse (see Table 1).

**Table 1:** **Values of sensitivity, specificity, and ROC area under the curve (AUC) by adherence assessment method**

		Adherence					Adherence + immunological outcome				
Adherence method	% cut-off	AUC	Accuracy	p-value	Sen	Spe	AUC	Accuracy	p-value	Sen	Spe
SHCS-AQ	NA	0.52	58.64	0.33	0.31	0.73	0.52	56.79	0.90	0.38	0.66
VAS	95	0.53	64.20	<0.0001	0.16	0.89	0.56	64.81	<0.01	0.27	0.84
Appointment keeping	80	0.53	62.11	0.01	0.25	0.81	0.59	64.60	0.43	0.42	0.76
Pharmacy refill	95	0.61	61.11	0.02	0.60	0.62	0.63	61.11	<0.0001	0.69	0.57
Pill count	90	0.53	44.03	<0.0001	0.79	0.26	0.53	44.10	<0.0001	0.82	0.25

Key: SHCS-AQ = Swiss HIV Cohort Study Adherence Questionnaire;

VAS = Visual Analog Scale; AUC = Area under the Curve; ROC = Receiver Operating Characteristic; Sen = Sensitivity; Spe = Specificity; NA = not applicable.

### Factors significantly associated with virological and immunological outcome

At recruitment, 13% of patients who were on treatment for more than four months had detectable viral load. This rate increased to 34% of all patients after the one-year follow-up. Two of 15 patients who started ART at recruitment still had a detectable viral load after one year. The odds of virological failure for various predictors are shown in Table 2. Patients who had a detectable viral load did not differ significantly from those with an undetectable viral load in terms of socio-demographic characteristics, except with respect to distance travelled to the centre: those with undetectable viral load lived closer, six (2 - 8) km as compared to seven (4.5 - 8.5) km in the subset with detectable viral load (p = 0.04). Patients with detectable viral load were more significantly taking triomune, a fixed combination of stavudine, lamivudine, and nevirapine, than those with undetectable viral load (p = 0.05). Detectable viral load at study entry, immunological failure at one year, and poor pharmacy refill adherence were other significant predictors of detectable viral load at the end of the study. Having a drug holiday and baseline CD4 T lymphocytes count had a borderline significance trend (see Additional file 4). The median IQR CD4 T lymphocytes count at last visit was 379 (255 - 541) cells/μl. Twenty-eight (17.3%) patients had CD4 T lymphocytes count less than 200 cells/μl.

**Table 2:** **Predictors of one year of follow-up virological and immunological failure by univariate logistic regression**

	Virological failure				Immunological failure	
	> 400 copies/ml		> 1,000 copies/ml			
Predictor	OR (95% CI)	p-value	OR (95% CI)	p-value	OR ( 95% CI)	p-value
Age per 1 year older	1 (0.96 - 1.03)	0.8	0.97 (0.93 - 1.02)	0.24	0.92 (0.85 - 0.98)	0.02
Being able to recall diagnosis date	0.39 (0.2 - 0.75)	0.01	0.3 (0.14 - 0.63)	<0.01	0.31(0.1 - 0.87)	0.03
Being on nevirapine regimen	2.17 (1.06 - 4.64)	0.04	2.76 (1.18 - 7.28)	0.03	1.3 (0.46 - 4.28)	0.64
Being on efavirenz regimen	0.48 (0.22 - 0.98)	0.05	0.37 (0.14 - 0.88)	0.03	0.79 (0.24 - 2.27)	0.68
Being on stavudine regimen	1.99 (1.03 - 3.89)	0.04	2.23 (1.06 - 4.83)	0.04	1.71 (0.62 - 4.94)	0.3
Body mass index per unit	1.06 (1.01 - 1.12)	0.03	1.02 (0.96 - 1.07)	0.53	0.97 (0.87 - 1.05)	0.5
Weight per 1 kg higher	1.03 (1 - 1.06)	0.03	1.02 (0.99 - 1.05)	0.13	0.99 (0.95 - 1.03)	0.65
Having baseline CD4 T lymphocytes count < 200 cell/μl	1.47 (0.7 - 3.03)	0.3	2.9 (1.32 - 6.35)	0.01	2.9 (1.02 - 8.17)	0.04
Therapy duration	8.67 (1.47 - 66.9)	0.02	8.75 (1.35 - 80.87)	0.03	2 (0.2 - 19.79)	0.53
Being on triomune regimen	2.07 (1.07 - 4.04)	0.03	2.31 (1.1 - 5)	0.03	1.76 (0.64 - 5.08)	0.28
Undetectable viral load at baseline	0.46 (0.21 - 1.01)	0.05	0.29 (0.13 - 0.68)	<0.01	0.23 (0.08 - 0.68)	0.01
Ever having drug holidays	3.32 (1.06 - 11.29)	0.04	5.2 (1.56 - 18.4)	0.01	2.46 (0.54 - 10.72)	0.23
SHCS-AQ adherence	0.83 (0.41 - 1.72)	0.61	0.78 (0.36 - 1.76)	0.54	0.7 (0.25 - 2.14)	0.51
VAS adherence (>95%)	0.65 (0.25 - 1.68)	0.36	0.54 (0.2 - 1.53)	0.22	0.21 (0.07 - 0.69)	0.01
Appointment adherence >80%)	0.68 (0.31 - 1.5)	0.33	0.65 (0.28 - 1.57)	0.32	1.28 (0.39 - 5.8)	0.71
Pharmacy refill adherence (>95%)	0.41 (0.21 - 0.8)	0.01	0.26 (0.12 - 0.57)	<0.01	1.23 (0.45 - 3.55)	0.69
Pill count adherence (>90%)	1.86 (0.62 - 5.5)	0.26	1.9 (0.56 - 5.78)	0.27	0.66 (0.04 - 3.7)	0.7

Key: SHCS-AQ = Swiss HIV Cohort study Adherence questionnaire; VAS = Visual analog scale; OR = odds ratio; CI = confidence interval.

In univariate LR analysis, odds of virological failure were determined. The study suggests that, being able to recall the diagnosis date, being on an efavirenz-containing regimen, having a pharmacy refill adherence of > 95% and having an undetectable viral load at baseline were significantly associated with lower likelihood of virological failure. On the contrary, having a baseline CD4 T lymphocytes count less than 200 cells/μl, being on a triomune, nevirapine, or stavudine regimen, having a higher body weight or body mass index (BMI), longer therapy duration, and having drug holidays were predictors of poor virological outcome (see Table 2). Adherence measured by the self-report methods VAS (>95%) and SHCS-AQ, appointment keeping (>80%) and pill count (>90%) (each time the optimum% cut-off was from Table 1) were not predictive of virological outcome. Older age, undetectable viral load at baseline, and VAS adherence were associated with lower likelihood of immunological failure, whereas having a baseline CD4 T lymphocytes count of less than 200 cells/μl was associated with immunological failure. According to the WHO definition of immunological failure, 17 of 162 (10.5%) patients had immunological failure at one year after recruitment.

**Figure 2:**
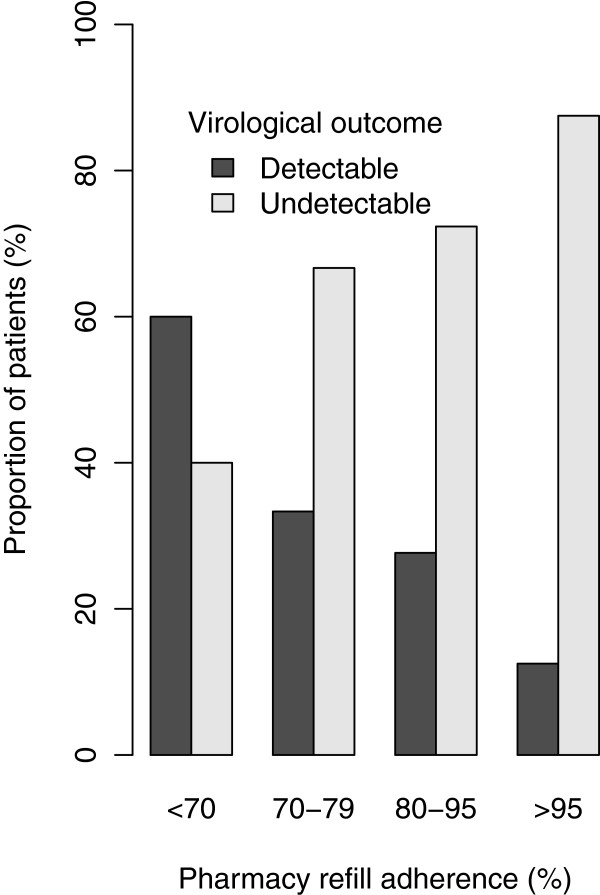
**Virological outcome in association with different pharmacy refill adherence rates.** Lowest viral load detection limit was 400 copies/ml.

Pharmacy refill was the only adherence measurement that was significantly associated with 12-month virological outcome (see Table 2 and Table 3). The proportion of patients with undetectable viral load increased with increasing pharmacy refill adherence, and was highest in patients with over 95% adherence (see Figure 2). Moreover, patients with detectable viral load had significantly lower pharmacy refill adherence with 91.9% (80 - 99.4%), as compared to 98.8% (91.1 – 100%) in patients with undetectable viral load (p = 0. 003). Of the 17 patients with immunological failure, 10 had a pharmacy refill adherence of > 95%.

**Table 3:** **Independent predictors of virological and immunological failure**

	> 400 copies/ml		>1,000 copies/ml		Immunological failure	
Predictor	AOR (95% CI)	p-value	AOR (95% CI)	p-value	AOR (95% CI)	p-value
Pharmacy refill adherence	0.45 (0.21 - 0.92)	0.03	0.24 (0.09 - 0.59)	<0.01	0.19 (0.04 - 0.81)	0.02
Being on triomune regimen	2.39 (1.16 - 5.07)	0.02	3.32 (1.36 - 8.64)	0.01	2.09 (0.66 - 7.07)	0.22
Having baseline CD4 T lymphocytes count < 200 cell/μl	1.59 (0.65 - 3.93)	0.31	3.91 (1.38 - 11.59)	0.01	2.05 (0.58 - 7.01)	0.25
Having baseline undetectable viral load	0.33 (0.12 - 0.87)	0.03	0.23 (0.07 - 0.7)	0.01	0.37 (0.1 - 1.35)	0.13
Being able to remember diagnosis date	0.36 (0.17 - 0.75)	0.01	0.23 (0.09 - 0.57)	<0.01	0.27 (0.07 - 0.84)	0.03
Age per 1 year older	1.01 (0.97 - 1.05)	0.71	1 (0.95 - 1.05)	0.94	0.97 (0.89 - 1.04)	0.37
Weight per 1 kg higher	1.05 (1.01 - 1.08)	0.01	1.05 (1.01 - 1.1)	0.01	0.99 (0.94 - 1.04)	0.65

Key: AOR (CI) = Adjusted odds ratio (confidence interval).

The sensitivity results indicated that the pharmacy refill adherence has the highest AUC and best balance between sensitivity and specificity, as compared with other adherence measurements (see Table 1 and Additional file 5). At a cut-off of 95%, refill adherence covers the highest AUC in the ROC curve and significantly predicts virological failure. The proportion decreased in a dose response fashion in lower adherence cut-off groups (see Figure 2). Increasing adherence was associated with increased rates of undetectable viral load.

### Multiple cross validation models to predict virological failure

Fifty independent runs of 10-fold MCV were executed, using different input sets and feature selection methods (see Table 4 and Additional file 6). MCV univariate models using a dichotomized adherence variable gave AUC and accuracy that were not much different than the univariate evaluation in Table 1 and Additional file 3, although sensitivity was consistently lower. A univariate logistic regression fitted on actual numeric (instead of optimized cut-off) pharmacy refill adherence yielded an AUC of 0.64 and accuracy of 68.73 (see Additional file 6). This was slightly better than with the optimal cut-off analysis shown in Table 1, but sensitivity was worse. A full LR model combining all variables in boost stepwise gave AUC of 0.64, which was higher than all the univariate adherence models, except the pharmacy refill adherence using continuous numeric values. The non-linear methods (decision trees and random forests) did not show improvement over the LR (see Table 4).

**Table 4:** **Performance of full variable model against the null models using over 10-fold cross validation repeated 50 times to predict virological failure**

Model	Goodness-of-fit [avg (st.dev)] with respect to failure			
	Area under roc	Accuracy	Sensitivity	Specificity
Majority class (null model)	0.50 (0.00)	66.07 (2.79)	0.00 (0.00)	1.00 (0.00)
LR on all variables with AIC stepwise	0.62 (0.14)	66.79 (10.10)	0.37 (0.20)	0.82 (0.12)
LR on all variables with boost stepwise	0.64 (0.14)	67.77 (9.82)	0.39 (0.20)	0.83 (0.11)
Random Forest on all variables	0.59 (0.15)	67.80 (9.11)	0.28 (0.19)	0.88 (0.10)
Decision tree on all variables	0.55 (0.12)	65.72 (8.46)	0.21 (0.21)	0.89 (0.13)

In multivariate analysis, having pharmacy refill adherence of < 95%, having a baseline CD4 T lymphocytes count < 200 cells/μl, being unable to recall the diagnosis date, and weight per 1 kg higher remained significant in predicting virological failure (see Table 3). Only pharmacy refill adherence and being able to recall diagnosis were significant predictors of immunological outcome.

### Kaplan-Meier and Cox model for immunological recovery

**Figure 3:**
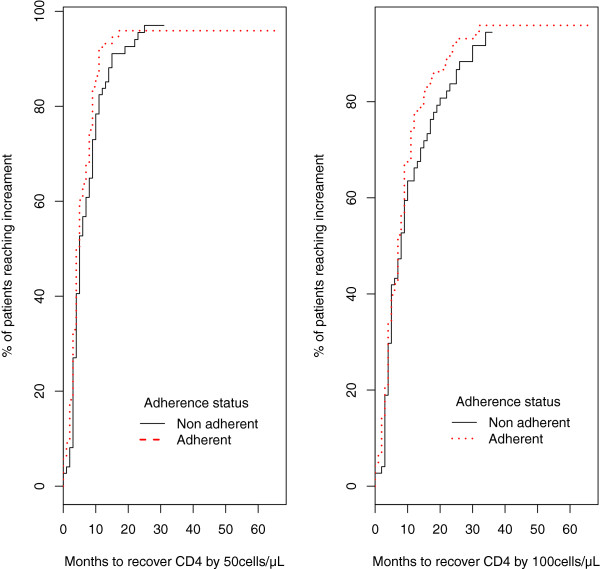
**Kaplan Meier survival curves showing the time to recover immunologically by 50 or 100 cells/μL at pharmacy refill at adherence cut-off of 95%.**

Kaplan Meier survival and Cox regression analysis was performed with months as the time unit, in order to assess the time taken by patients to recover CD4 T lymphocytes count by at least 50 or 100 cells/μl (see Figure 3). Patients who had a pharmacy refill adherence of < 95% recovered 50 and 100 cells/μl in a median (95% confidence interval) of five (4 - 8) and eight (5 - 10) weeks respectively. Whereas, patients who were > 95% refill adherent recovered in 4.5 (4 - 6) and seven (6 - 9) weeks respectively. Although the median time to reach the increment was longer in the non-adherent group, this difference did not reach significance (log rank p = 0.15 and 0.23 for increment of 50 and 100 cells/μl respectively). However, when pharmacy refill adherence was combined with age, gender, and CD4 T lymphocytes count at baseline, a Cox regression analysis resulted in significant differences (Wald test p < 0.01).

## Discussion

The aims of this study were to evaluate the correlation and validation of adherence with virological outcome in RLS. In RLS, viral load monitoring, the preferred method for evaluating treatment outcome, is hindered by financial and technical constraints, and the widely-used indicators of response to therapy, as well as clinical and immunological outcome monitoring, are confounded by poor sensitivity. The best measurement of adherence, along with other affordable and easily-obtained variables, could be used in RLS as a proxy for virological failure. The best model using such variables to predict virological failure may be useful to flag patients for viral load measurements, as a cheaper alternative than universal viral load follow-up. This study was carried out as an exploratory study using convenient sampling, and can, therefore, serve to inform larger studies on pharmacy refill adherence as a predictor of virological treatment response.

Four measures of adherence used in a RLS were validated against a one-year virological response as a “gold standard”. Although it is widely agreed that adherence plays an important role in the ART outcome, there is no standardized measure of adherence agreed upon in RLS. Each adherent measurement method has inherent weaknesses in various settings.

There was a high proportion of patients with virological failure (34%) in the study, despite high self-reporting adherence of > 95% in up to 87% of patients. Factors significantly associated with virological failure at the one-year of follow-up were having pharmacy refill adherence of < 95%, having baseline CD4 T lymphocytes count < 200 cells/μl, being on triomune-, stavudine-, or nevirapine-containing regimen, not being able to recall HIV diagnosis date, a unit increase in body weight even after correcting for age, and a baseline viral load. The association between the uses of triomune may be related to the negative correlation of both stavudine and nevirapine individually, rather than the fixed-dose therapy itself. These two drug components are both independently associated with virological failure after one year of therapy.

Of the adherence measurements, only pharmacy refill adherence was positively correlated with the virological response in a dose response relationship per each increasing adherence rate stratified category. The dose response relationship between pharmacy refill adherence and virological outcome has also been observed in previous studies [10, 23, 33]. Increasing the virological failure threshold to a viral load > 1,000 copies/mL led to an increase of sensitivity and specificity, confirming previous observation [34]. While this is to be expected, given that lack of adherence will result in lack of virus control, the more relevant clinical cut-off remains the lower cut-off of 400 copies/ml, which was more related to residual replication of partially-resistant virus under drug selective pressure. Similar factors predicted the immunological outcome. The correlation of pharmacy refill adherence and virological outcome has been shown previously [35]. Moreover, pharmacy refill showed a promising applicability, given its high performance in multiple validation regression models, balanced sensitivity and specificity compared to self-reports, appointment keeping, or pill count methods. Superiority of pharmacy refill adherence over self-report has been shown in a number of studies in RRS [10, 33, 36] and RLS [23, 37]. Generally, lower sensitivity and higher specificity values were noted for adherence measurement to predict virological failure, as compared to predicting virological success. This indicates that adherence measurement is more likely to detect adherent persons who are likely to have a good virologic outcome, as compared to identifying failing patients. Combining all variables in a multiple cross validation-full logistic regression model did not improve much over the pharmacy refill univariate model. The non-linear models test for interactions among variables, and especially random forests perform better if the variables are interdependent. Since the linear regression model performed better than the non-linear models, this indicates that the variables analysed are behaving independently with respect to the outcome. Thus, the power of pharmacy refill to predict virological failure is independent of other patient variables.

Even though in this study the optimal cut-off for pharmacy refill was 95%, the dose response relationship shown in the comparison of virological outcome and grouped pharmacy refill adherence suggests that ARV regimens suppress viral load even at moderate adherence of > 70%. This may be related to current regimens that are more potent, and, thus, may sufficiently sustain complete viral suppression and avoid HIVDR, especially among patients initiating ART [7, 17, 38].

Pharmacy refill adherence measurement has a potential to be used in RLS [24, 39]. In most RLS undertaking special ART scale-up programs, patients receiving free ARV supplies are obliged to return to the same centre on a monthly basis to obtain their medication refills, which allows implementation of this method. Furthermore, where the pharmacies have a computerized database, the pharmacy refill adherence measurement becomes feasible, practical, and guaranteed [39].

There are some limitations to the application of pharmacy refill adherence. First is the assumption that the drugs refilled are always consumed. There is no way to tell if the patient sold, shared, or dumped the medications after a refill. For that matter, this method is a mere proxy for adherence. A second drawback is inability to detect or predict all patients with viral rebound, given the relatively-low sensitivity for predicting virological failure (around 60%). Moreover, with this method, it is not possible to discriminate patterns of adherence, such as treatment interruption, which may be more risky for non-nucleoside reverse transcriptase inhibitor resistance than a run of occasionally missed doses [6, 40]. An additional drawback is when an individual can acquire medication refills from multiple pharmacies outside the regular clinic. However, in most RLS, patients have to return to the same clinic for their monthly ARV refills [7], and, thus, pharmacy refill is a feasible adherence measurement.

In this study, self-report measures did not predict virological outcome. Self-reported adherence has not been consistently validated in RLS [23]. In some studies that evaluated self-report, it was found to be less sensitive [37, 41]. The unreliable nature of self-report stems from participants’ beliefs and fears about how the information provided to clinicians or adherence counsellors would be used. Thus, accuracy of self-reported adherence may be undermined if patients fear that reporting non-adherence could have negative consequences in their healthcare provisions, which are more relevant in RLS. In addition, clinic-based pill counts could not predict virologic outcome. Generally, clinic-based pill counts performed poorly in other studies, also [42]. Unannounced pill counts conducted in homes have been closely associated with virological suppression [7, 43], the development of HIVDR [7], and progression to AIDS [44], but are less feasible. This study found no association between appointment keeping and viral load. Authors who found a better correlation [45] suggested that monitoring of clinic attendance may be an objective and effective measure, and could be a useful adjunct to an adherence measure, such as pill counting in RLS. Human resource constraints in these settings do not allow pill counts or other time-consuming measures to be taken. Therefore, monitoring clinic attendance and acting on missed appointments may be an effective proxy measure [45].

Furthermore, in a Cox regression model, after accounting for age, gender, and immunological status, the groups of patients having pharmacy refill > 95% were more likely to recover immunologically in a relatively-shorter period than the non-adherent group. The cross validation after fitting the adherence in logistic regression indicate that adherence estimates combined with other variables performs better in predicting the virological outcome than adherence alone. These results indicated that a model combining only adherence and immunological response performs equally well, even better than using adherence alone. This model is important in routine clinical practice because immunological outcome is easily available, whereas other variables may not be so easily and routinely available to use for predicting therapy response.

The study findings are limited by a small sample size obtained by convenient sampling of individuals who were initiating or continuing ART within the three months period of recruitment at the study site.

## Conclusions

Of the adherence methods investigated in this study, pharmacy refill method had the best performance in predicting virological failure. The performance improved when pharmacy refill adherence was combined with immunological response. The study found, this combination to have a reasonable sensitivity (around 70%) and specificity (around 60%) to predict virological failure. This combination could be useful to flag patients at risk for virological failure. It appeared to be more reliable than immunological and clinical response alone and was cost-saving. Thus, it had the potential for a wider application in ART follow-up in RLS. When combined with even more variables, such as treatment and demographic characteristics in the full logistic regression model, the prediction still improved, however this may become less straightforward and may, thus, lose applicability in RLS where doctors have very little time to devote to individual patients*.*

## Electronic supplementary material

Additional file 1: **Socio-demographic characteristics of participants included versus excluded.** Socio-demographic characteristics of participants. (DOCX 20 KB)

Additional file 2: **Values of sensitivity, specificity, ROC curve area under the curve (AUC) by adherence assessment method at different cut-off points predicting virological failure (viral load > 1,000 copies/ml) and immunological failure, using adherence measurement.** Values of sensitivity, specificity and AUC by predicting virological failure or immunological failure using adherence measurements. (DOCX 24 KB)

Additional file 3: **Values of sensitivity, specificity, ROC curve AUC by adherence assessment method at different cut-off points predicting virological failure (viral load < 400 copies/ml) at individual time points of adherence follow-up measurement.** Description of data: Four time points 1 - 4 and the overall adherence outcome is shown per adherence measurement method. Key: SHCS-AQ = Swiss HIV Cohort Study Adherence Questionnaire; VAS = Visual Analog Scale; AUC = Area Under the Curve; ROC = Receiver Operating Characteristic; Sen = Sensitivity; Spe = Specificity; CI = confidence interval; PPV = positive predictive value; NPV = negative predictive value; DR = detection rate; DP = detection prevalence. AUC noted as “NA” indicated a situation when one of the two groups (adherent or non-adherent) was too small to calculate sensitivity and specificity. (XLSX 21 KB)

Additional file 4: **Socio-demographic characteristics of patients by detectable versus undetectable viral load at one year of follow-up.** Comparison of social-demographic characteristics grouped by detectable versus undetectable viral load. (DOCX 19 KB)

Additional file 5: **ROC curve using sensitivity and specificity for predicting virological failure at different pharmacy refill adherence threshold cut-off points between 50 and 100.** Note an increase in the area under the curve with increasing cut-off until optimum at 95%. ROC curve for predicting virological failure at different pharmacy refill adherence cut-off points. (EPS 5 KB)

Additional file 6: **Performance of and adherence univariate models versus full variable-null model using over 10-fold cross validation, repeated 50 times to predict virological failure (>400 copies/ml).** Good-ness of fit of predicting virological failure using univariate versus full variable model. (DOCX 19 KB)

## Abbreviations

AIC: Akaike information criterion
AIDS: Acquired immunodeficiency syndrome
ART: Antiretroviral therapy
ARV: Antiretroviral
AUC: Area under the curve
CD4: Cluster of differentiation 4
CI: Confidence interval
CTC: Care and Treatment Centre
EFV: Efavirenz
HIV: Human immunodeficiency virus
HIVDR: HIV drug resistance
IQR: Interquartile range
LR: Logistic regression
MEMS: Medication event monitoring system
MCV: Multiple cross validation
NVP: Nevirapine
OR: Odds ratio
RLS: Resource-limited settings
ROC: receiver operating characteristic
RRS: resource-rich settings
SHCS-AQ: Swiss HIV Cohort study-adherence questionnaire
VAS: Visual analog scale.
