# Supplementary material for: Pharmacy refill adherence outperforms self-reported methods in predicting HIV therapy outcome in resource-limited settings
Source: BMC Public Health. 2014 Oct 4;14:1035. doi: 10.1186/1471-2458-14-1035 (PMC4194413; doi:10.1186/1471-2458-14-1035)
Supplement: Supplementary file 1 — Additional file 1: Socio-demographic characteristics of participants included versus excluded. Socio-demographic characteristics of participants. (DOCX 20 KB) [file 12889_2014_7132_MOESM1_ESM.docx]

**Additional file 1**

**Socio-demographic characteristics of participants included versus excluded.**

|  | All   (N=220) | Included (N=162) | Excluded   (N=58) | p-value |
| --- | --- | --- | --- | --- |
| Characteristic | N (%)  ^*^N (IQR) | N (%)  ^*^N (IQR) | N (%)  ^*^N (IQR) |  |
| Age (years) | 39 (34 - 47) ^*^ | 40 (35 - 47) | 37 (33 - 44) | 0.11 |
| Distance to the CTC (km) | 7 (3 - 8) ^*^ | 6 (2 - 8) ^*^ | 7 (4 - 8) ^*^ | 0.14 |
| Marital status |  |  |  | 0.47 |
| Married | 100 (46.9) | 68 (43.9) | 32 (55.2) |  |
| Single/Divorced/widow (er) | 113 (53.1) | 87 (56.1 | 26 (44.8) |  |
| Gender |  |  |  | 0.36 |
| F | 143 (63.8) | 100 (61.7) | 43 (69.4) |  |
| M | 81 (36.2) | 62 (38.3) | 19 (30.6) |  |
| Education |  |  |  | 0.37 |
| Up to grade 7 | 162 (74.6) | 114 (72.6) | 48 (80) |  |
| >Grade 7 | 55 (25.4) | 43 (27.4) | 12 (20) |  |
| Income (€ per month) |  |  |  | 0.47 |
| None | 76 (34.5) | 58 (36.2) | 18 (30) |  |
| <50 | 105 (47.7) | 72 (45) | 33 (55) |  |
| 50-250 | 37 (16.8) | 29 (18.1) | 8 (13.3) |  |
| 250-500 | 2 (0.9) | 1 (0.6) | 1 (1.7) |  |
| Religion |  |  |  | 0.11 |
| Christian | 102 (49.8) | 66 (45.2) | 36 (61) |  |
| Muslim | 102 (49.8) | 79 (54.1) | 23 (39) |  |
| Other | 1 (0.5) | 1 (0.7) | 0 (0) |  |
| Duration since diagnosis (months) | 27 (18 - 43.5)^*^ | 27 (19 - 41) ^*^ | 27 (16 - 47) ^*^ | 0.81 |
| Year of ART start | 2008 (2007 - 2009) ^*^ | 2008 (2007 - 2009) ^*^ | 2008 (2007 - 2009) ^*^ | 0.39 |
| Duration of ART at recruitment (months) | 24 (16 - 36) ^*^ | 24 (16 - 35) ^*^ | 23 (12 - 37) ^*^ | 0.36 |
| Being on atripla regimen | 19 (8.5) | 18 (11.1) | 1 (1.6) | 0.04 |
| Being on a once-daily single tablet regimen | 21 (9.4) | 20 (12.3) | 1 (1.6) | 0.03 |
| Being on triomune regimen | 104 (46.4) | 75 (46.3) | 29 (46.8) | 1 |
| WHO HIV disease staging |  |  |  | 0.68 |
| I | 12 (5.4) | 7 (4.3) | 5 (8.1) |  |
| II | 40 (17.9) | 28 (17.3) | 12 (19.4) |  |
| III | 150 (67) | 111 (68.5) | 39 (62.9) |  |
| IV | 22 (9.8) | 16 (9.9) | 6 (9.7) |  |
| CD4 T lymphocytes count at recruitment (cell/µl) | 293 (200.5 - 466.5) ^*^ | 293 (198 - 469) ^*^ | 286 (217 - 443) ^*^ | 0.63 |
| Viral load (log) | 2.6 (2.6 - 2.6) | 2.6 (2.6 - 2.6) | 2.6 (2.6 - 2.7) | 0.28 |
| Undetectable viral load at baseline | 175 (78.1) | 129 (79.6) | 46 (74.2) | 0.34 |
| Immunological failure at 1 year | 197 (87.9) | 145 (89.5) | 52 (83.9) | 0.35 |
| Overall VAS adherence | 100 (97.5 - 100) ^*^ | 100 (98.3 - 100) ^*^ | 100 (96.7 - 100) ^*^ | 0.24 |
| Overall appointment adherence | 100 (88.3 - 100) ^*^ | 100 (84.3 - 100) ^*^ | 100 (89.6 - 100) ^*^ | 0.74 |
| Pharmacy refill adherence | 96.6 (84.9 - 100) ^*^ | 96.8 (86 - 100) ^*^ | 94.1 (77.1 - 100) ^*^ | 0.48 |
| Overall pill count adherence | 84.6 (74.5 - 90.6) ^*^ | 83.6 (74 - 89.9) ^*^ | 87 (78.6 - 92.3) ^*^ | 0.06 |
| Number of CD4 T lymphocytes count measurements | 8 (6 - 11) ^*^ | 8 (6 - 11) ^*^ | 7 (4 - 11) ^*^ | 0.03 |
| Duration since ART scale-up (months) | 53 (40.5 - 61) ^*^ | 53 (41 - 60) ^*^ | 54 (39 - 65) ^*^ | 0.35 |
| Ever missing appointment | 64 (28.6) | 46 (28.4) | 18 (29) | 1 |
| Simply forgetting to take ART | 51 (22.8) | 33 (20.4) | 18 (29) | 0.23 |
| Drug holidays | 21 (28.8) | 17 (27.4) | 4 (36.4) | 0.81 |
| Health condition improved after ART | 143 (66.5) | 109 (69.9) | 34 (57.6) | 0.22 |
| Alcohol consumption | 110 (51.4) | 80 (51.6) | 30 (50.8) | 0.79 |
| Disclosing status to relatives | 87 (41.4) | 61 (40.1) | 26 (44.8) | 0.65 |

Key: IQR=interquartile range; ART = antiretroviral; ART = antiretroviral therapy; VL = viral load; CTC = Care and Treatment Centre; VAS = Visual analog scale; N= number of patients; ^*^Values are given as N (IQR)
