# Supplementary material for: Pharmacy refill adherence outperforms self-reported methods in predicting HIV therapy outcome in resource-limited settings
Source: BMC Public Health. 2014 Oct 4;14:1035. doi: 10.1186/1471-2458-14-1035 (PMC4194413; doi:10.1186/1471-2458-14-1035)
Supplement: Supplementary file 2 — Additional file 2: Values of sensitivity, specificity, ROC curve area under the curve (AUC) by adherence assessment method at different cut-off points predicting virological failure (viral load > 1,000 copies/ml) and immunological failure, using adherence measurement. Values of sensitivity, specificity and AUC by predicting virological failure or immunological failure using adherence measurements. (DOCX 24 KB) [file 12889_2014_7132_MOESM2_ESM.docx]

**Additional file 2**

**Values of sensitivity, specificity, ROC curve area under the curve (AUC) by adherence assessment method at different cut-off points predicting virological failure (viral load > 1,000 copies/ml) and immunological failure, using adherence measurement.**

|  |  | Virological failure (> 1000 copies/ml) | | | | |  | Immunological failure | | | | |
| --- | --- | --- | --- | --- | --- | --- | --- | --- | --- | --- | --- | --- |
| Adherence method | % cut-off^*^ | AUC | Accuracy | p-value | Sen | Spe |  | AUC | Accuracy | p-value | Sen | Spe |
| SHCS-AQ | NA | 0.53 | 63.58 | 2.98E-01 | 0.32 | 0.73 |  | NA | 68.52 | 8.83E-05 | 0.35 | 0.72 |
| VAS | 90 | 0.55 | 77.16 | 1.92E-05 | 0.14 | 0.96 |  | NA | 90.74 | 1.21E-01 | 0.35 | 0.97 |
| **VAS** | 95 | 0.54 | 72.84 | 2.37E-02 | 0.19 | 0.89 |  | NA | 83.95 | 5.56E-01 | 0.35 | 0.9 |
| VAS | 100 | 0.55 | 61.73 | 1.58E-02 | 0.43 | 0.67 |  | NA | 65.43 | 1.87E-07 | 0.53 | 0.67 |
| Appointment | 50 | 0.54 | 77.64 | 1.34E-06 | 0.11 | 0.98 |  | NA | 85.09 | 6.62E-02 | 0 | 0.95 |
| Appointment | 55 | 0.53 | 76.4 | 1.19E-05 | 0.11 | 0.96 |  | NA | 83.85 | 1.70E-01 | 0 | 0.94 |
| Appointment | 60 | 0.54 | 76.4 | 5.00E-05 | 0.14 | 0.95 |  | NA | 85.09 | 3.07E-01 | 0.12 | 0.94 |
| Appointment | 65 | 0.54 | 75.16 | 2.76E-04 | 0.14 | 0.94 |  | NA | 83.85 | 5.56E-01 | 0.12 | 0.92 |
| Appointment | 70 | 0.52 | 70.81 | 4.11E-02 | 0.16 | 0.87 |  | NA | 78.26 | 4.99E-01 | 0.12 | 0.86 |
| Appointment | 75 | 0.55 | 69.57 | 5.68E-01 | 0.27 | 0.82 |  | NA | 72.05 | 3.69E-02 | 0.12 | 0.79 |
| **Appointment** | 80 | NA | 68.32 | 7.79E-01 | 0.27 | 0.81 |  | NA | 72.05 | 1.71E-02 | 0.18 | 0.78 |
| Appointment | 85 | NA | 63.98 | 6.94E-01 | 0.27 | 0.75 |  | NA | 67.7 | 1.43E-03 | 0.18 | 0.74 |
| Appointment | 90 | NA | 62.11 | 4.42E-01 | 0.27 | 0.73 |  | NA | 67.08 | 3.55E-04 | 0.24 | 0.72 |
| Appointment | 95 | NA | 60.25 | 1.69E-01 | 0.3 | 0.69 |  | NA | 63.98 | 4.69E-05 | 0.24 | 0.69 |
| Appointment | 100 | 0.52 | 59.01 | 1.94E-02 | 0.38 | 0.65 |  | NA | 60.25 | 1.09E-06 | 0.29 | 0.64 |
| Refill | 50 | 0.53 | 77.78 | 2.38E-07 | 0.08 | 0.98 |  | NA | 87.65 | 1.39E-02 | 0.06 | 0.97 |
| Refill | 55 | 0.57 | 79.01 | 3.65E-06 | 0.16 | 0.98 |  | NA | 87.65 | 1.18E-01 | 0.18 | 0.96 |
| Refill | 60 | 0.6 | 80.25 | 9.90E-06 | 0.22 | 0.98 |  | NA | 86.42 | 2.86E-01 | 0.18 | 0.94 |
| Refill | 65 | 0.61 | 80.86 | 1.63E-05 | 0.24 | 0.98 |  | NA | 85.8 | 4.04E-01 | 0.18 | 0.94 |
| Refill | 70 | 0.6 | 79.01 | 3.16E-04 | 0.24 | 0.95 |  | NA | 83.95 | 8.45E-01 | 0.18 | 0.92 |
| Refill | 75 | 0.59 | 76.54 | 1.50E-02 | 0.27 | 0.91 |  | NA | 81.48 | 5.84E-01 | 0.24 | 0.88 |
| Refill | 80 | 0.62 | 76.54 | 1.44E-01 | 0.35 | 0.89 |  | NA | 79.01 | 1.23E-01 | 0.29 | 0.85 |
| Refill | 85 | 0.62 | 72.22 | 7.66E-01 | 0.43 | 0.81 |  | NA | 72.22 | 1.04E-03 | 0.35 | 0.77 |
| Refill | 90 | 0.64 | 70.37 | 1.12E-01 | 0.51 | 0.76 |  | NA | 66.67 | 2.46E-05 | 0.35 | 0.7 |
| **Refill** | 95 | 0.66 | 63.58 | 2.78E-06 | 0.7 | 0.62 |  | NA | 52.47 | 1.75E-10 | 0.41 | 0.54 |
| Refill | 100 | 0.6 | 52.47 | 1.75E-10 | 0.73 | 0.46 |  | NA | 41.36 | 6.32E-15 | 0.47 | 0.41 |
| Pill | 50 | NA | 72.96 | 2.52E-04 | 0.03 | 0.93 |  | NA | 86.79 | 3.83E-01 | 0.13 | 0.94 |
| Pill | 55 | NA | 73.58 | 1.19E-03 | 0.09 | 0.92 |  | NA | 84.91 | 8.38E-01 | 0.13 | 0.92 |
| Pill | 60 | NA | 71.7 | 1.71E-02 | 0.11 | 0.89 |  | NA | 83.02 | 7.00E-01 | 0.2 | 0.9 |
| Pill | 65 | NA | 69.18 | 2.53E-01 | 0.17 | 0.84 |  | NA | 79.25 | 8.17E-02 | 0.27 | 0.85 |
| Pill | 70 | NA | 67.3 | 6.77E-01 | 0.2 | 0.81 |  | NA | 77.36 | 1.24E-02 | 0.33 | 0.82 |
| Pill | 75 | NA | 62.26 | 2.45E-01 | 0.29 | 0.72 |  | NA | 71.07 | 1.90E-05 | 0.47 | 0.74 |
| Pill | 80 | 0.51 | 56.6 | 1.75E-03 | 0.4 | 0.61 |  | NA | 61.64 | 3.87E-09 | 0.53 | 0.63 |
| Pill | 85 | NA | 46.54 | 5.85E-08 | 0.51 | 0.45 |  | NA | 46.54 | 3.14E-14 | 0.53 | 0.46 |
| **Pill** | 90 | 0.51 | 36.48 | 2.00E-19 | 0.77 | 0.25 |  | NA | 30.19 | 2.00E-19 | 0.8 | 0.25 |
| Pill | 95 | NA | 25.16 | 2.00E-19 | 0.86 | 0.08 |  | NA | 17.61 | 2.00E-19 | 0.93 | 0.1 |
| Pill | 100 | NA | 21.38 | 2.00E-19 | 0.97 | 0 |  | NA | 10.06 | 2.00E-19 | 1.00 | 0.01 |

Key: SHCS-AQ= Swiss HIV Cohort Study Adherence Questionnaire; VAS=Visual Analog Scale; AUC=Area Under the Curve; ROC = Receiver Operating Characteristic; Sen = Sensitivity; Spe = Specificity.

The method in bold was considered optimum, given the AUC sensitivity and specificity.

^*^The cut-off range tested for VAS (only 90, 95, and 100) adherence was different from the other measurement, since its values were skewed close to the mean (98.25); the median IQR was 100 (98.33 - 100).
