# Supplementary material for: Pharmacy refill adherence outperforms self-reported methods in predicting HIV therapy outcome in resource-limited settings
Source: BMC Public Health. 2014 Oct 4;14:1035. doi: 10.1186/1471-2458-14-1035 (PMC4194413; doi:10.1186/1471-2458-14-1035)
Supplement: Supplementary file 4 — Additional file 4: Socio-demographic characteristics of patients by detectable versus undetectable viral load at one year of follow-up. Comparison of social-demographic characteristics grouped by detectable versus undetectable viral load. (DOCX 19 KB) [file 12889_2014_7132_MOESM4_ESM.docx]

**Additional File 4**

**Socio-demographic characteristics of patients by detectable versus undetectable viral load at one year of follow-up.**

|  | All  (N=162) | Detectable VL (N=55) | Undetectable VL (N=107) | p-value |
| --- | --- | --- | --- | --- |
| Characteristic | N (%)  *median (IQR) | N (%)  *median (IQR) | N (%)  *median (IQR) |  |
| Age (years) | 40 (35 - 47) * | 40 (34.5 - 47) * | 39 (35 - 47) * | 0.87 |
| **Distance to the CTC (km)** | **6 (2 - 8)** | **7 (4.5 - 8.5)** | **6 (2 - 8)** | **0.04** |
| Marital status |  |  |  | 0.21 |
| Married | 68 (43.9) | 21 (41.2) | 47 (45.2) |  |
| Single/Divorced/widow (er) | 87 (56.1) | 30 (58.8) | 57 (54.8) |  |
| Gender |  |  |  | 0.60 |
| Female | 100 (61.7) | 36 (65.5) | 64 (59.8) |  |
| Male | 62 (38.3) | 19 (34.5) | 43 (40.2) |  |
| Education |  |  |  | 0.58 |
| Up to grade 7 | 114 (72.6) | 36 (66.7) | 78 (75.7) |  |
| > Grade 7 | 43 (27.4) | 18 (33.3) | 24 (24.3) |  |
| Income (€ per month) |  |  |  | 0.86 |
| None | 58 (36.2) | 20 (36.4) | 38 (36.2) |  |
| <50 | 72 (45) | 26 (47.3) | 46 (43.8) |  |
| 50-250 | 29 (18.1) | 9 (16.4) | 20 (19) |  |
| 250-500 | 1 (0.6) | 0 (0) | 1 (1) |  |
| Religion |  |  |  | 0.75 |
| Christian | 66 (45.2) | 23 (46.9) | 43 (44.3) |  |
| Muslim | 79 (54.1) | 26 (53.1) | 53 (54.6) |  |
| Other | 1 (0.7) | 0 (0) | 1 (1) |  |
| Duration since diagnosis (months) | 27 (19 - 41) * | 27 (20 - 42) * | 27 (18 - 39) * | 0.86 |
| Duration of ART at recruitment (months) | 24 (16 - 35) * | 22 (15.5 - 35) * | 24 (17.5 - 34.5) * | 0.27 |
| Year of ART start | 2008 (2007 - 2009) * | 2008 (2007 - 2009) * | 2008 (2007 - 2008.5) * | 0.30 |
| Being on once-daily single tablet regimen | 20 (12.3) | 4 (7.3) | 16 (15) | 0.25 |
| **Being on triomune regimen** | **75 (46.3)** | **32 (58.2)** | **43 (40.2)** | **0.05** |
| WHO HIV disease staging at recruitment |  |  |  | 0.89 |
| I | 7 (4.3) | 2 (3.6) | 5 (4.7) |  |
| II | 28 (17.3) | 8 (14.5) | 20 (18.7) |  |
| III | 111 (68.5) | 39 (70.9) | 72 (67.3) |  |
| IV | 16 (9.9) | 6 (10.9) | 10 (9.3) |  |
| CD4 T lymphocytes count at recruitment | 293 (198 - 469) * | 270 (165.5 - 437) * | 306 (216.5 - 495.5) * | 0.09 |
| Number of CD4 T lymphocytes count measurements | 8 (6 - 11) * | 8 (6 - 11) * | 8 (6 - 11) * | 0.68 |
| **Immunological failure at 1 year** | **17 (10.5)** | **10 (18.2)** | **7 (6.5)** | **0.04** |
| **Undetectable viral load at baseline** | **129 (79.6)** | **39 (70.9)** | **90 (84.1)** | **0.02** |
| Overall VAS adherence | 100 (98.3 - 100) * | 100 (97.5 - 100) * | 100 (98.3 - 100) * | 0.25 |
| Overall appointment adherence | 100 (84.3 - 100) | 100 (83.4 - 100) | 100 (84.3 - 100) * | 0.37 |
| **Pharmacy refill adherence** | **96.8 (86 - 100)** | **91.9 (80 - 99.4) *** | **98.8 (91.1 - 100) *** | **<0.01** |
| Overall pill count adherence | 83.6 (74 - 89.9) | 84 (76.2 - 88.7) * | 83.5 (72.8 - 90.2) * | 0.70 |
| Duration since ART scale up (months) | 53 (41 - 60) * | 55 (40.5 - 61) * | 52 (42 - 59.5) * | 0.31 |
| Ever missing appointment | 46 (28.4) | 17 (30.9) | 29 (27.1) | 0.75 |
| Simply forgetting to take ART | 33 (20.4) | 13 (23.6) | 20 (18.7) | 0.59 |
| Drug holidays | 17 (27.4) | 11 (40.7) | 6 (17.1) | 0.08 |
| Perceiving health condition improvement after ART | 109 (69.9) | 40 (72.7) | 69 (68.3) | 0.52 |
| Alcohol consumption | 80 (51.6) | 30 (54.5) | 50 (50) | 0.72 |
| Disclosing status to relatives | 61 (40.1) | 20 (39.2) | 41 (40.6) | 1 |

Key: IQR = interquartile range; ART = antiretroviral; ART = antiretroviral therapy; VL = viral load; CTC = Care and Treatment Centre; VAS = Visual analog scale.

Significantly different proportions with p-value < 0.05 are shown in **bold**.
