# Supplementary material for: Pharmacy refill adherence outperforms self-reported methods in predicting HIV therapy outcome in resource-limited settings
Source: BMC Public Health. 2014 Oct 4;14:1035. doi: 10.1186/1471-2458-14-1035 (PMC4194413; doi:10.1186/1471-2458-14-1035)
Supplement: Supplementary file 6 — Additional file 6: Performance of and adherence univariate models versus full variable-null model using over 10-fold cross validation, repeated 50 times to predict virological failure (>400 copies/ml). Good-ness of fit of predicting virological failure using univariate versus full variable model. (DOCX 19 KB) [file 12889_2014_7132_MOESM6_ESM.docx]

**Additional File 6**

**Performance of and adherence univariate models versus full variable-null model using over 10-fold cross validation, repeated 50 times to predict virological failure (> 400 copies/ml).**

The Null model of reference is the model that predicted the most frequent class (majority class), which was the virological failure at VL > 400 copies per ml. The reference class 1 (class 1) had 0 % sensitivity (true positive rate) and 100 % specificity (true negative rate). Univariate logistic regression models with stepwise function (AIC).

Variables included were either dichotomized (at % cut-off), numeric, or quartile values of adherence measurement indicated.

The following univariate models did not perform better from the null model; appointment (numeric), pill count (numeric), VAS (<100 %), VAS (quartiles), appointment (< 100 %), appointment (< 95 %), appointment (< 90 %), pharmacy refill (< 100 %), pill count (< 90 %), pill count (< 85 %), pill count (quartiles). See Table 1 for adherence and sensitivity definitions in the context of the study. Degrees of freedom for t-test were 50.

| Model | Goodness-of-fit [avg (st.dev)] with respect to failure | | | |
| --- | --- | --- | --- | --- |
|  | Area under roc | Accuracy | Sensitivity | Specificity |
| Majority class (null model) | 0.50 (0.00) | 66.07 (2.79) | 0.00 (0.00) | 1.00 (0.00) |
| SHCS-AQ (0 or 1) | 0.48 (0.12) | 66.07 (2.79) | 0.00 (0.00) | 1.00 (0.00) |
| VAS (numeric) | 0.55 (0.12) | 65.52 (3.60) | 0.01 (0.04) | 0.99 (0.04) |
| Pharmacy refill (numeric) | 0.64 (0.14) | 68.73 (6.34) | 0.14 (0.14) | 0.97 (0.06) |
| VAS (<95 %) | 0.54 (0.07) | 64.01 (4.32) | 0.04 (0.08) | 0.95 (0.07) |
| VAS (<90 %) | 0.54 (0.06) | 67.92 (5.07) | 0.09 (0.11) | 0.98 (0.04) |
| Appointment (quartiles) | 0.51 (0.13) | 64.87 (5.04) | 0.00 (0.03) | 0.98 (0.07) |
| Pharmacy refill (<90 %) | 0.60 (0.12) | 63.12 (6.81) | 0.05 (0.13) | 0.93 (0.15) |
| Pharmacy refill (quartiles) | 0.59 (0.14) | 64.99 (5.52) | 0.01 (0.03) | 0.98 (0.09) |
| Pill count (<95 %) | 0.54 (0.08) | 63.48 (4.93) | 0.06 (0.10) | 0.93 (0.08) |
